# Genetic modulation of oxytocin sensitivity: a pharmacogenetic approach

**DOI:** 10.1038/tp.2015.163

**Published:** 2015-10-27

**Authors:** F S Chen, R Kumsta, F Dvorak, G Domes, O S Yim, R P Ebstein, M Heinrichs

**Affiliations:** 1 Department of Psychology, Laboratory for Biological and Personality Psychology, University of Freiburg, Freiburg, Germany; 2 Department of Psychology, University of British Columbia, Vancouver, BC, Canada; 3 Department of Genetic Psychology, Faculty of Psychology, Ruhr-University Bochum, Bochum, Germany; 4 Graduate School of Decision Sciences, University of Konstanz, Konstanz, Germany; 5 Department of Economics, Chair of Applied Research in Economics, University of Konstanz, Konstanz, Germany; 6 Department of Psychology, National University of Singapore, Singapore, Singapore; 7 Freiburg Brain Imaging Center, University Medical Center, University of Freiburg, Freiburg, Germany

## Abstract

Intranasal administration of the neuropeptide oxytocin has been shown to influence a range of complex social cognitions and social behaviors, and it holds therapeutic potential for the treatment of mental disorders characterized by social functioning deficits such as autism, social phobia and borderline personality disorder. However, considerable variability exists in individual responses to oxytocin administration. Here, we undertook a study to investigate the role of genetic variation in sensitivity to exogenous oxytocin using a socioemotional task. In a randomized, double-blind, placebo-controlled experiment with a repeated-measures (crossover) design, we assessed the performance of 203 men on an emotion recognition task under oxytocin and placebo. We took a haplotype-based approach to investigate the association between oxytocin receptor gene variation and oxytocin sensitivity. We identified a six-marker haplotype block spanning the promoter region and intron 3 that was significantly associated with our measure of oxytocin sensitivity. Specifically, the TTCGGG haplotype comprising single-nucleotide polymorphisms rs237917–rs2268498–rs4564970–rs237897–rs2268495–rs53576 is associated with increased emotion recognition performance under oxytocin versus placebo, and the CCGAGA haplotype with the opposite pattern. These results on the genetic modulation of sensitivity to oxytocin document a significant source of individual differences with implications for personalized treatment approaches using oxytocin administration.

## Introduction

The neuropeptide oxytocin has a key role in the regulation of complex social cognition and behaviors, including mammalian pair bonding, maternal behavior, recognition of conspecifics and responses to social stress.^1, 2^ Human studies have documented a number of effects of intranasally administered oxytocin on social behavior and social cognition.^3, 4, 5^ For instance, a single dose of intranasal oxytocin increases trusting behavior,^6^ attention to the eye region of faces^7, 8^ and the accuracy with which individuals interpret emotional information from eyes.^9^ Oxytocin also reduces subjective and physiological responses to social stress and enhances the stress-buffering effect of social support.^10^ Neuroimaging studies have shown that one of the mechanisms through which oxytocin influences social cognition is by attenuating amygdala responses to social stimuli such as emotional faces.^11, 12, 13, 14^

There has also been considerable interest in the therapeutic potential of oxytocin for the treatment of mental disorders characterized by social functioning deficits such as autism,^15, 16, 17, 18, 19^ social phobia^20, 21^ and borderline personality disorder.^22^ Whereas the results of virtually all of these studies suggest that oxytocin has the potential to improve social cognition and social behavior in healthy individuals and patient populations, there is considerable variability in individual responsiveness to oxytocin administration, with some individuals showing strong behavioral effects in response to intranasal oxytocin and others seeming to show minimal or no effects.^3^ These findings suggest that oxytocin administration may interact with pre-existing interindividual variation that influences the efficacy of oxytocin signaling in the central nervous system. Specifically, genetic variation influencing the number, distribution or functioning of oxytocin receptors may influence oxytocin sensitivity. Research investigating the genetic modulation of sensitivity to oxytocin administration has implications not only for basic research but also implications for clinicians who would like to predict which patients may or may not benefit from oxytocin administration, and in general may help to refine personalized oxytocin-based therapies for mental disorders.

Initial studies using a molecular genetic approach suggest that common variations in the human oxytocin system are relevant for social behavior.^23^ Specifically, variation in the human oxytocin receptor gene (*OXTR*) has been associated in some studies to susceptibility for mental disorders characterized by social deficits such as autism spectrum disorder^24^ (although the specific single-nucleotide polymorphism(s) (SNP(s)) and allele(s) linked to greater susceptibility have varied depending on ethnicity and other sample characteristics).^25, 26, 27, 28^ In addition, variants of *OXTR* have been linked to individual differences in psychological and physiological response patterns to stress and social information,^29, 30^ as well as to differences in general sociobehavioral phenotypes. These include prosocial behavior,^31^ empathy,^32^ trust^33^ and sensitive parenting.^34^ Structural and functional imaging studies have shown associations between *OXTR* SNPs and morphometric alterations as well as differences in activity of neural circuits involved in the processing of social information and negative affect. These imaging studies suggest that genetic variation of *OXTR* influences limbic circuitry involving the amygdala, the hypothalamus and the cingulate gyrus.^35, 36, 37, 38^

Given the associations that have been documented between *OXTR* variability and social behavior, and given that oxytocin exerts its function via the oxytocin receptor, *OXTR* variability is a prime candidate for explaining individual differences in response to exogenous oxytocin administration. To test this possibility, we conducted a randomized, double-blind, placebo-controlled experiment with a repeated-measures (crossover) design. We assessed each participant's performance on an emotion recognition task previously shown to be sensitive to oxytocin administration,^39^ both under oxytocin and placebo. This within-subject design provides a measure of each individual's socioemotional sensitivity to oxytocin administration.

Whereas most association studies so far have focused on single SNPs, we investigated 23 SNPs across the *OXTR* gene region and took a haplotype-based approach in our analyses. In addition to allowing us to capture more genetic information than single SNP analysis,^40^ the haplotype approach—which by definition jointly tests multiple SNPs for association—allows us to avoid the conundrum of multiple testing, which can result in false-positive results. As has been noted by other researchers,^41^ divergent results between studies may sometimes have arisen from unrecognized haplotype effects because these studies were limited to investigation of individual SNPs. In addition, questionable results may arise from disregarding linkage disequilibrium (LD) of the tested polymorphism, an effect preventable by haplotype analysis (see Binder *et al.*^42^ for further discussion of haplotype analysis).

## Materials and methods

### Subjects

We tested 207 men between 19 and 30 years of age (*M*=23.5 years, s.d.=2.7 years) of Central European descent from the student population of the University of Freiburg, Germany, who were recruited through on-campus advertisements. The sample size was determined on the basis of recent research demonstrating effects of common variants of *OXTR* on behavior.^43, 44, 45, 46, 47^ Participants were pre-screened to ensure that they were fluent in German, were not suffering from mental or physical illnesses and were not taking drugs or medication. Students studying psychology were excluded from our sample. All participants gave written, informed consent before the start of the first experimental session and received a total of 40 Euro for participation in the two sessions. The study was approved by the institutional review board of the University of Freiburg.

### Procedure

Each participant was tested twice, with a 1-week gap between testing sessions. All sessions took place in the afternoon and lasted ~2 h. Participants were tested in a laboratory in groups of 12–16, with each participant seated at a computer in an individual cubicle. Dividing walls prevented visual contact between the participants, and participants were instructed not to speak to each other during the testing sessions.

#### Substance administration

After completing a questionnaire to assess baseline mood (the Multidimensional Mood Questionnaire^48^), participants received either oxytocin (Syntocinon-Spray, Novartis, Basel, Switzerland) or placebo intranasally in a randomized, double-blind, crossover design. The placebo included all of the same ingredients as the oxytocin spray except for the neuropeptide. Under the supervision of the experimenter, participants self-administered 24 IU of the substance (three puffs into each nostril in alternating order).^10^ After a loading period of 45 min, the participants completed a second Multidimensional Mood Questionnaire to assess their mood.

#### Emotion recognition task

Participants subsequently completed a task based closely on one used by Lischke *et al.*^39^ to assess their ability to recognize emotions from dynamic facial expressions. Previous research suggests that this task is sensitive to oxytocin administration: participants who received oxytocin identified emotions more quickly and at a lower intensity level than participants who received placebo.^39^ In each trial, a photo of an individual displaying a neutral expression gradually morphed into a photo of the same individual displaying a happy, angry, fearful or sad expression. The participant's task was to press a button when he recognized the emotion being displayed. Participants were instructed to respond as quickly and accurately as possible. As soon as the participant responded, the morphing procedure stopped and the image was replaced by a prompt asking the participant to indicate which of the four emotions he had detected (see Supplementary Information for details).

#### Non-social control task

To test whether any observed effects would be specific to social stimuli, participants also completed a control task for the recognition of non-social stimuli (cars) after the emotion recognition task. Using Winmorph 3.01 (http://www.debugmode.com/winmorph/), grayscale images of a common car model (a small hatchback) were morphed into images of target cars (four exemplars each of the car types: Smart car, box truck, pickup truck and VW Beetle). As in the emotion recognition task, participants were instructed to press a button as soon as they recognized the target car type. Before the 16 test trials, participants completed four practice trials. The test trials appeared in one of two pseudorandomized testing orders. For each trial, reaction time and accuracy were recorded.

During the second session, a saliva sample was collected from each participant (Oragene 500-OG kit, DNA Genotek, Ottawa, Ontario, Canada) for genotyping. Participants were also asked at the end of the second session whether they thought they had received oxytocin or placebo in that session.

### SNP selection and genotyping

The goal of this study was to associate variation of *OXTR* with oxytocin sensitivity. In order to capture the maximum amount of genetic information, we chose 23 SNPs across the OXTR gene for subsequent haplotype-based analyses. Sixteen SNPs were selected on the basis of an association study with SNP coverage across *OXTR*.^26^ Seven additional SNPs were selected; rs53576 was included because a large number of studies have shown significant associations between this SNP and sociobehavioral phenotypes.^23^ SNP rs2268498, located in the promoter region, was included because of its presumed functional significance.^49^ In order to increase coverage of the 5′ region, a likely region of regulatory activity, four additional SNPs compatible with the multiplex assay were selected from SNP databases. This selection procedure is a standard *a priori* approach to haplotype testing, where not a single SNP but rather SNPs across a gene region and their pattern of LD are taken into account.

DNA was extracted from saliva in Oragene collection vials by desalting procedure following the manufacturer's protocol. SNP genotyping was performed on the Sequenom MassArray platform using iPLEX Gold chemistry (Sequenom, San Diego, CA, USA). Primers and multiplexes were designed using the Sequenom's MassArray Assay Design v4.0 Software. Multiplex PCR was performed using iPlex Gold reagents in 5-μl reactions as recommended by the manufacturer. Three hundred eighty-four-well PCR plates were used for PCR amplification on a GeneAmp 9700 thermocycler (Applied Biosystems, Waltham, MA, USA), and the products were spotted onto 384-well SpectroCHIP arrays (Sequenom) using the Nanodispenser RS1000 (Sequenom). Spotted SpectroCHIPs were then fired in a MassArray Analyzer 4 Matrix-Assisted Laser Desorption/Ionization-Time Of Flight mass spectrometer. Procedures used were as in the manufacturer's protocol. Data analyses were performed on Sequenom's Typer Software Module, and ambiguous genotype calls were manually confirmed. Out of the 23 genotyped SNPs (see Supplementary Table 1), 7 were not in Hardy–Weinberg equilibrium and 2 SNPs had a minor allele frequency below 5%. Genotyping failed for SNP rs237894. Therefore, 13 SNPs were included in the association test (see Results).

### Statistics

We analyze data from all participants (*N*=203) who completed the emotion recognition task in both sessions and for whom genotyping was successful. Accuracy of emotion recognition and reaction time were recorded for each trial. Accuracy was near ceiling (*M*=88%, s.d.=9%) and is thus not considered in the following analyses. Reaction times were averaged across all accurate trials.

Preliminary analyses showed a main effect of time, indicating that reaction times improved between the first and second sessions for all groups. Therefore, reaction times were standardized (*z*-transformed) separately for each session (1 and 2). In order to obtain a measure for oxytocin sensitivity, a difference score was calculated (subtracting standardized reaction times under oxytocin from standardized reaction times under placebo).

Haplotype analyses were performed with version 3.1.6 of the program UNPHASED.^50^ Associations between haplotypes and the quantitative trait oxytocin sensitivity were tested with the *individual haplotype test* implemented in UNPHASED. This option gives individual tests for each haplotype in turn, and tests for a difference between a haplotype and all the others pooled together. The outcome variable was our measure for oxytocin sensitivity, that is, the difference in reaction time between oxytocin and placebo. Using a sliding window approach, seven to two marker haplotypes were tested. Only haplotypes with a frequency of >2.5% were analyzed. In order to correct for multiple testing, the permutation test option as provided in UNPHASED was used. Permutation test correction was performed using 1000 iterations (random permutations) and was applied to correction of global *P*-values (the *P*-value generated by UNPHASED as an overall test of each block of haplotypes).

In order to confirm the results obtained using UNPHASED, which used the difference score as an outcome measure, a General Linear Model for repeated measures, with reaction time under oxytocin and placebo as repeated variables, haplotype as the between-subject factor, and order of substance administration as a covariate was computed (SPSS Version 20, IBM, Armonk, NY, USA). Assumptions of normality, equality of variance in the groups being compared, and sphericity were checked and met by the data. In all analyses, two-tailed *P*-values are reported.

## Results

**Figure 1:**
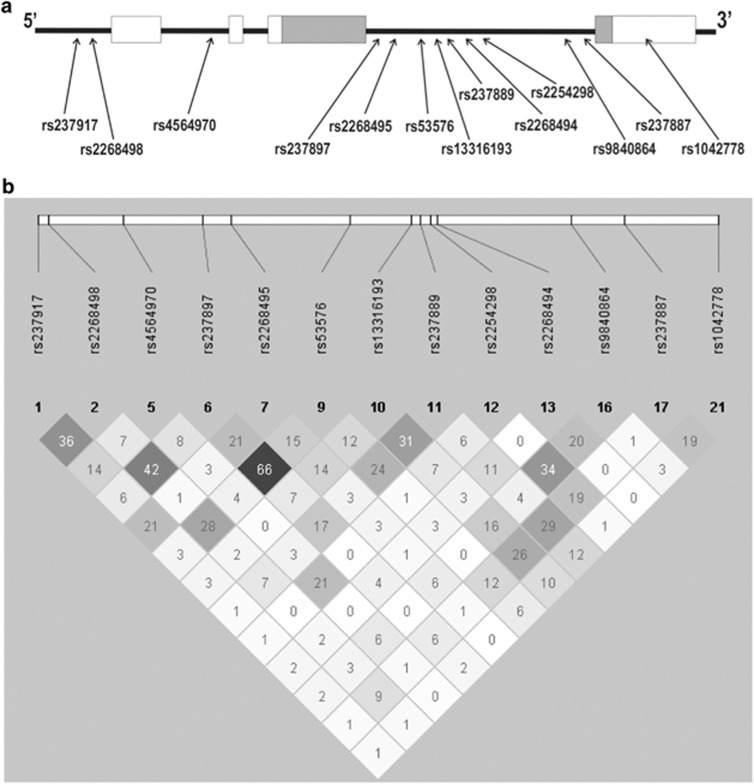
Upper panel **a** shows the location of the single-nucleotide polymorphisms (SNPs) along the *OXTR* gene. Lower panel **b** shows the linkage disequilibrium map of the *OXTR* SNPs genotyped in our sample produced by Haploview.

The assayed SNPs, their location along the *OXTR* genomic region and their relationship to the exon/intron boundaries of the gene are shown in the upper part of Figure 1. The lower part shows the LD map of the genotyped SNPs as depicted by Haploview.

Haplotype analyses revealed significant associations between oxytocin sensitivity and haplotypes of marker sizes six to two (Table 1). A more detailed analysis of the six-marker block showed that two haplotypes differed significantly from the remaining ones: CCGAGA and TTCGGG (with frequencies of 7.1% and 28.3%, respectively; see Table 2).

**Figure 2:**
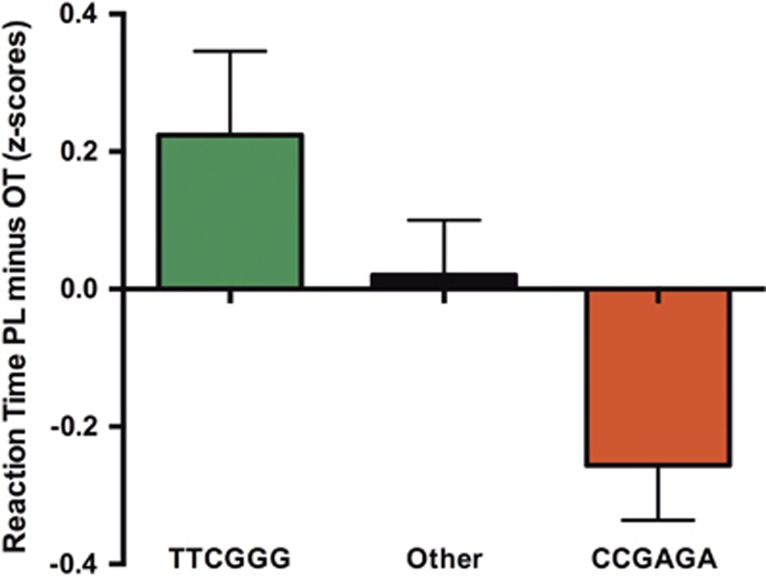
Graph shows relative oxytocin sensitivity across groups for the six-marker haplotype block rs237917–rs2268498–rs4564970–rs237897–rs2268495–rs53576. Oxytocin sensitivity was indexed in terms of emotion recognition speed under oxytocin compared with placebo. Higher values indicate faster reaction times under oxytocin.

In order to specify the direction of effects, we compared oxytocin sensitivity scores (based on the emotion recognition speed under oxytocin versus placebo; see Materials and methods) among three groups: carriers of the two significant haplotype groups (TTCGGG and CCGAGA) and the group comprising the remaining haplotype combinations (*Other* group). As shown in Figure 2, the two significant six-marker haplotypes showed opposing effects. Both carriers of the TTCGGG haplotype and carriers of the CCGAGA haplotype showed differential sensitivity to oxytocin relative to the *Other* group. Specifically, carriers of the TTCGGG haplotype recognized emotions more quickly and at a lower intensity level under oxytocin compared with placebo; CCGAGA carriers showed the opposite pattern. When all genotype groups were combined, there was no main effect of oxytocin on emotion recognition speed.

The results obtained using UNPHASED, which used the difference score as the outcome measure, were confirmed with a general linear model for repeated measures, with reaction time under oxytocin and placebo as repeated variables, six-marker haplotype as the between-subject factor and order of substance administration as a covariate (haplotype by substance interaction: F_2,186_=5.80; *P*=0.004).

With regard to the blocks of smaller window sizes, with very few exceptions, the same allelic combinations (comprising subsets of the alleles in the six-marker haplotypes) drove the significant associations (see Supplementary Table 2; detailed results for the other haplotype windows can be found in Supplementary Table 3).

**Figure 3:**
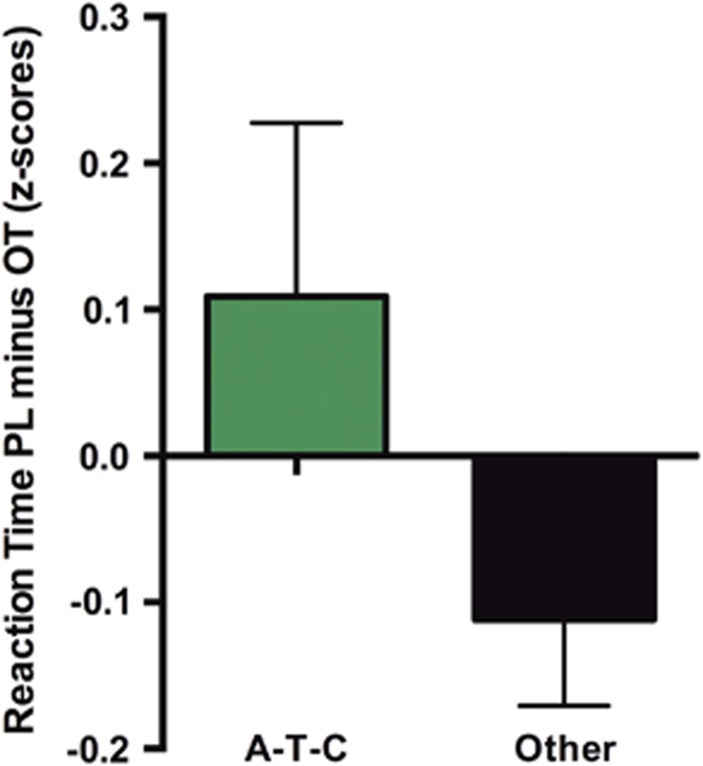
Graph shows relative oxytocin sensitivity across groups for the three-marker haplotype block rs2254298–rs2268494–rs9840864. Oxytocin sensitivity was indexed in terms of emotion recognition speed under oxytocin compared with placebo. Higher values indicate faster reaction times under oxytocin.

There is one additional three-marker haplotype in intron 3 comprising SNPs rs2254298–rs2268494–rs9840864 showing significant results (Figure 3). As shown in Table 3, the A-allele of rs2254298, which also shows a nominally significant association on the single SNP level, falls exclusively on the significant haplotype. Furthermore, the six SNPs that make up the significant six-marker haplotype and additionally rs2254298 were significantly associated with sensitivity to oxytocin on their own (see bottom of Table 1), with rs237897 surviving Bonferroni correction for 13 tested SNPs.

### Non-social control condition and additional analyses

General Linear Model analyses showed no haplotype by substance interactions (F_2,180_=1.52; *P*=0.22) under the non-social control condition, which used cars that morphed from a ‘neutral' model into one of four other models as stimuli, supporting the view that the effects of oxytocin are specific for social stimuli.

Finally, as a group, participants were not able to tell whether they had received oxytocin or placebo (the accuracy rate of 53% did not differ from the chance level of 50%, *Z*=0.85, *P*=0.39). Participants' mood was not affected by receiving oxytocin versus placebo (that is, changes in Multidimensional Mood Questionnaire scores from pre- to post-spray application did not differ by the substance group, all *P*>0.20).

## Discussion

There are several lines of evidence implicating the central oxytocin system in the etiology of mental disorders characterized by social deficits. This is supported by genetic studies linking SNPs and haplotypes of *OXTR* to individual differences in social behavior. Furthermore, several of these SNPs have been associated with structural and functional changes in human brain regions involved in a regulatory circuit of socioemotional information processing. The discovery that neuropeptides can be non-invasively delivered to the brain in humans has raised considerable interest in the therapeutic potential of oxytocin for the treatment of disorders characterized by social functioning deficits.^3, 4^

Here, we investigated whether sensitivity to oxytocin administration is influenced by genetic variation of *OXTR*. We identified a six-marker haplotype block spanning the promoter region and intron 3 that was significantly associated with our measure of oxytocin sensitivity, emotion recognition performance under oxytocin versus placebo. Specifically, we identified two haplotypes that were differentially associated with oxytocin sensitivity: the TTCGGG haplotype comprising SNPs rs237917–rs2268498–rs4564970–rs237897–rs2268495–rs53576 is associated with increased emotion recognition performance under oxytocin versus placebo, and the CCGAGA haplotype with the opposite pattern. It is of note that the A allele of rs53576, previously associated with phenotypes including reduced empathy, lower trust, reduced sensitivity to social support in the context of stress and less sensitive parenting, falls almost exclusively on the CCGAGA haplotype. In the present study, this haplotype was associated with decreased emotion recognition speed under oxytocin compared with placebo.

The functional consequences associated with this haplotype are currently unknown. Speculatively, it is possible that previously reported findings in the literature involving rs53576 are functionally explained by LD with the SNPs located in the regulatory region of *OXTR,* possibly influencing transcriptional efficiency. There is tentative evidence that promoter SNP rs2268498, associated with negative emotionality^49^ and moral judgments,^51^ influences OXTR gene regulation (referred to in Montag *et al.*^49^ as unpublished data). Detailed functional *in vitro* characterization of *OXTR* promoter SNPs, including rs2268498, is warranted.

Although a number of other studies have used single SNP approaches to examine the effect of oxytocin intranasal administration on social cognition, we are not aware of the specific use of haplotype analysis in experiments that involve intranasal oxytocin administration. There are studies that have used haplotype analysis to examine association between the oxytocin receptor, behavior and psychopathology. For instance, in a study by Lerer *et al.,*^26^ several OXTR haplotypes were associated with autism. Significant findings emerged for haplotypes comprising SNPs in intron 1 and the untranslated part of exon 4. Of note, associations were shown with haplotypes of several sizes including SNPs rs237897 and/or rs2254298. The first SNP is part of our six-marker haplotype, and rs2254298 is tagging the three-marker haplotype identified in the present study. Another haplotype-based analysis identified an association between social cognition and a haplotype consisting of rs11131149 and rs2254298.^52^ A third study showed that a haplotype comprising SNPs rs9840864 and rs2268494—the latter being part of the significant three-marker block in our study—was associated with anger and retaliation after betrayal.^47^ This shows some convergence between studies; however, as our study had a more extensive coverage of the 5′ region of the gene, and the other studies did not or only partially include the SNPs of our six-marker haplotype, the results cannot be directly compared.

Our results suggest that oxytocin administration facilitates emotion recognition in some individuals more than others. The fact that no main effect of oxytocin was observed in this study when all genotype groups were combined highlights the critical influence of individual difference factors in oxytocin administration effects. The ability to recognize the emotions of others is a building block for empathy and has been associated with prosocial behavior.^53^ Impairments in the ability to recognize emotions are also characteristic of several mental disorders involving social deficits, including autism^54^ and borderline personality disorder.^55^ Preliminary evidence suggests that oxytocin may indeed alleviate impairments in emotion recognition in some clinical samples.^8, 18, 56^ By identifying particular individuals who respond to oxytocin administration more strongly than others in the context of an emotion recognition task, the current study provides a first step toward predicting which individuals are likely to profit from oxytocin-based treatments intended to alleviate impairments in socioemotional functioning.

We chose the emotion recognition task in our study based on the fact that it has been shown to be sensitive to oxytocin administration.^39^ Furthermore, because it involves dynamic stimuli, this task arguably has more ecological validity than tasks involving static photos of faces. However, future research will be necessary to test the generalizability of our reported findings to other emotion recognition tasks, to other tasks relevant for empathy and prosocial behavior and to other types of settings and contexts.

Because previous results have suggested sex differences in associations between *OXTR* genotype and phenotype^57^ as well as in responses to intranasal oxytocin administration,^11, 58^ we decided to limit our current study to male participants. Follow-up studies testing women are necessary to examine the generalizability of our findings and are expected to be particularly informative, given the potential for sex differences in this domain. As with all genetic association studies, our findings should be replicated in an independent sample and with a larger sample size to establish the robustness of the effects we observed. Given that different directions of genotype–phenotype associations have been observed in different ethnicities,^25, 28^ future work should also test the effects reported here in other ethnic groups. Furthermore, the distribution of these haplotypes in samples with impairments in socioemotional functioning should be documented in future research. This information on haplotype distributions may help to elucidate some of the potential sources of these socioemotional impairments and may also help identify specific samples for which oxytocin-based therapies are more or less likely to be effective.

It is also likely that epigenetic modifications of *OXTR* influence oxytocin sensitivity.^59^ Functional studies have shown that differential methylation of a CpG island in the *OXTR* promoter seems to be functionally important for *OXTR* expression,^60^ and differences in the degree of methylation have been observed in childhood disorders characterized by impairments in social cognition, including autism^61^ and conduct problems concurrent with callous-unemotional traits.^62^

In addition to *OXTR*, other factors involved in the oxytocin pathway might influence oxytocin sensitivity. These include the gene for oxytocin (*OXT*; coding for the precursor protein oxytocin-neurophysin-I), the gene encoding the enzyme that metabolizes oxytocin, oxytocinase (human leucyl/cystinylaminopeptidase; *LNPEP*) as well as *CD38*, a key mediator of oxytocin brain release.^63, 64^ In the long term, future studies involving larger sample sizes may help to elucidate the role of these other genes, along with potential gene–gene interactions in oxytocin sensitivity.

The current study, which demonstrates genetic modulation of sensitivity to oxytocin administration, identifies one specific and systematic source of individual differences in response to oxytocin administration. The identification of this new haplotype block conferring differences in oxytocin-induced socioemotional behavior will not only help advance a basic scientific understanding of the human oxytocin system but may also improve the prediction of clinical outcomes of novel therapy approaches using oxytocin. More specifically, these results may help to bridge the insights from a pharmacogenetic approach to psychobiological therapy combining oxytocin administration and psychotherapy^4, 65, 66^ for genetically targeted subgroups of patients with social deficits across diagnostic categories of mental disorders (for example, autism spectrum disorder, social anxiety disorder and borderline personality disorder). More such personalized treatment strategies^67, 68^ are necessary to help fulfill the immense promise of translational success of oxytocin-based therapies.

## Figures and Tables

**Table 1:**
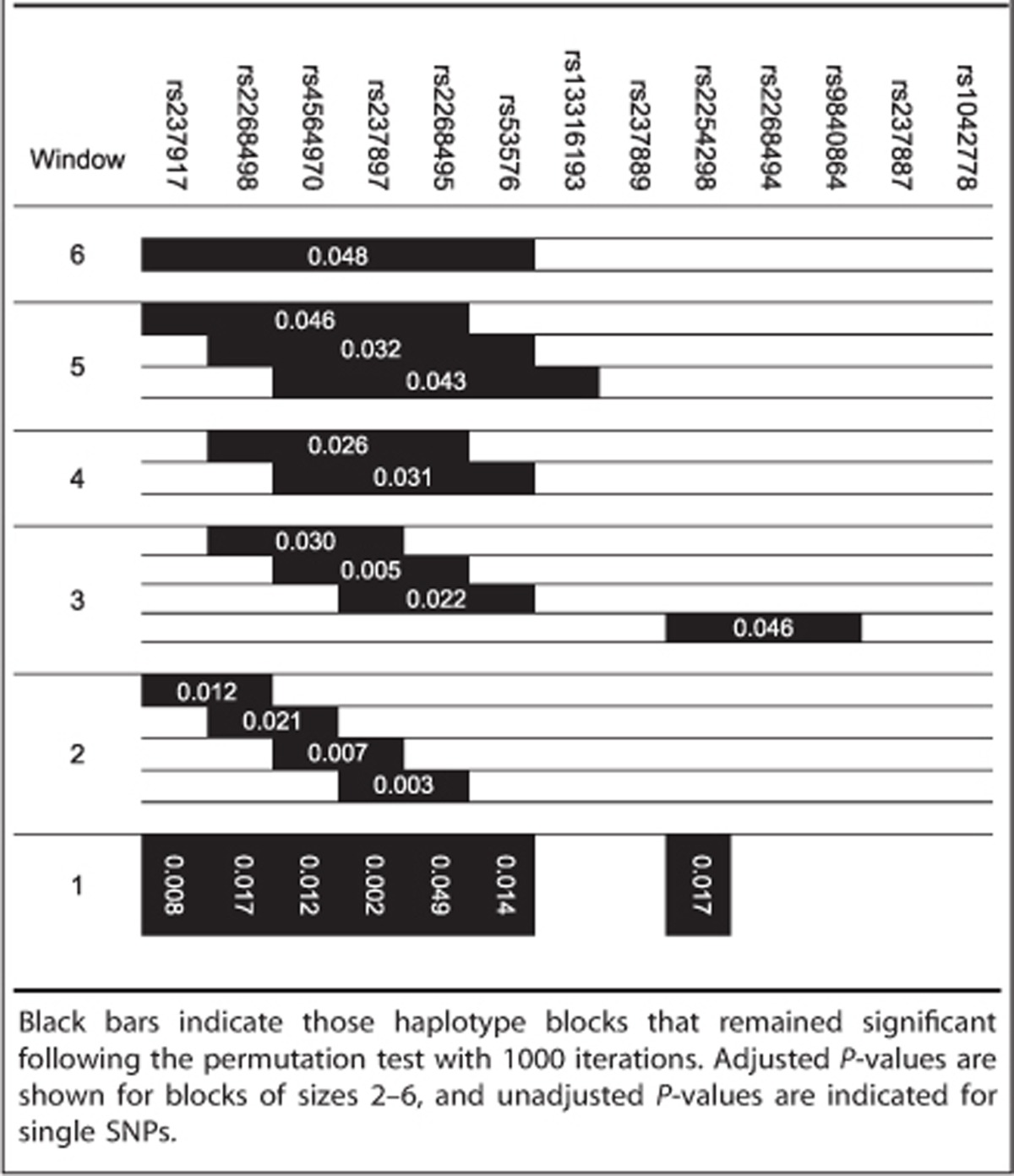
Haplotypes associated with oxytocin sensitivity

**Table 2:** Detailed analysis of 6-marker haplotype block rs237917–rs2268498–rs4564970–rs237897–rs2268495–rs53576

	*Haplotype*	*Frequency*	*X*^*2*^	P
	*Likelihood ratio X*^*2*^=*14.78, df=6, global* P-*value=0.022 (0.048)*			
	**C-C-G-A-G-A**	**0.283**	**7.483**	**0.006**
	C-T-G-G-G-G	0.218	0.127	0.720
	T-T-G-G-A-G	0.156	3.113	0.078
	C-C-G-A-G-G	0.074	0.083	0.774
	**T-T-C-G-G-G**	**0.071**	**6.681**	**0.010**
	C-C-G-G-A-G	0.066	0.877	0.349
	T-T-G-A-G-A	0.049	0.176	0.675

Abbreviation: df, degrees of freedom. Global *P*-value and permutation-corrected *P*-value (shown in brackets) for the six-marker haplotype window. Individual tests for each haplotype showed that two haplotypes (CCGAGA and TTCGGG) differed significantly (indicated in bold) from the remaining haplotypes.

**Table 3:** Detailed analysis of 3-marker haplotype block rs2254298–rs2268494–rs9840864

	*Haplotype*	*Frequency*	*X*^*2*^	P
	*Likelihood ratio* X^*2*^=*8.23, df=3, global* P-*value=0.042 (0.046)*			
	G-T-G	0.771	0.336	0.562
	**A-T-C**	**0.093**	**6.269**	**0.012**
	G-T-C	0.070	2.386	0.122
	G-A-C	0.060	0.151	0.697

Abbreviation: df, degrees of freedom. Global *P*-value and permutation-corrected *P*-value (shown in brackets) for the three-marker haplotype window that falls outside the region delineated by the six-marker window shown above. The ATC haplotype, tagged by the A allele of rs2254298, differs significantly (indicated in bold) from the remaining haplotypes.
