# Supplementary material for: Genetic modulation of oxytocin sensitivity: a pharmacogenetic approach
Source: Transl Psychiatry. 2015 Oct 27;5(10):e664–. doi: 10.1038/tp.2015.163 (PMC4930136; doi:10.1038/tp.2015.163)
Supplement: Supplementary Information [file tp2015163x1.pdf]

Supplementary Information

Emotion Recognition Task

Stimuli for the task were created using still images of three men and three women from the FACES database[1]. All of the images were converted into grayscale and rescaled to a standard size using Adobe Photoshop CS4 (Adobe Systems Inc., San Jose, CA, USA) and Matlab 7.7 (MathWorks Inc., Natick, MA, USA). Overall brightness and contrast were also normalized for all images. To hide hair and background, an elliptical mask (411 x 570 pixels) was overlaid over each image.

Using WinMorph 3.01 (http://www.debugmode/winmorph), facial images of individuals displaying neutral expressions were morphed into images of the same individuals displaying full-intensity emotional expressions (happy, angry, fearful, and angry). In total, images of three men and three women were converted into stimuli for 24 test trials (6 individuals x 4 emotions). Each trial consisted of a sequence of 100 still images that appeared to participants as a dynamic emotional expression transitioning smoothly from 0% to 100% intensity over 8 seconds.

Images were presented on a grey background and a 20” monitor (30.6 x 40.8 cm, resolution 1024 x 768 pixels) at a viewing distance of 55 cm. Before the 24 test trials, participants completed four practice trials, for which images from the FACES database of another individual displaying the four target emotions were used. The test trials appeared in one of two pseudo-randomized testing orders. For each trial, reaction time and accuracy were recorded.

1. Ebner NC, Riediger M, Lindenberger U. FACES--a database of facial expressions in young, middle-aged, and older women and men: development and validation. *Behav Res Methods* 2010; **42**:351-62.

Supplementary Table 1: 23 SNPs across the *OXTR* gene region were analyzed. 10 were excluded from further analyses because of deviation from Hardy-Weinberg Equilibrium (cut-off p<.05; SNPs 3, 14, 15,1 8, 19, 22, 23), minor allele frequency (MAF) below 5% (SNPs 4 & 20), or genotyping failure (SNP 8). SNPs included in the association test are shown in bold.

	rs number	chr position	Alleles	MAF	HWE p value	% Genotyped
**1**	**rs237917**	**8812699**	**C/T**	**0.296**	**0.149**	**96.0**
**2**	**rs2268498**	**8812411**	**T/C**	**0.470**	**0.956**	**99.0**
3	rs3806675	8811646	T/C	0.428	0.029	80.4
4	rs73132859	8811394	G/C	0.036	1.0	98.5
**5**	**rs4564970**	**8810408**	**G/C**	**0.106**	**0.175**	**99.5**
**6**	**rs237897**	**8808285**	**G/A**	**0.432**	**0.317**	**100.0**
**7**	**rs2268495**	**8807535**	**G/A**	**0.216**	**0.783**	**100.0**
8	rs237894	8806531	failed genotyping			
**9**	**rs53576**	**8804371**	**G/A**	**0.36**	**0.528**	**99.0**
**10**	**rs13316193**	**8802743**	**T/C**	**0.389**	**0.894**	**100.0**
**11**	**rs237889**	**8802483**	**C/T**	**0.346**	**0.182**	**94.5**
**12**	**rs2254298**	**8802228**	**G/A**	**0.106**	**0.175**	**99.5**
**13**	**rs2268494**	**8802046**	**T/A**	**0.056**	**0.921**	**99.5**
14	rs2268492	8800672	C/T	0.286	0.023	93.0
15	rs4686301	8798586	C/T	0.342	0.045	100.0
**16**	**rs9840864**	**8798477**	**G/C**	**0.218**	**0.604**	**99.0**
**17**	**rs237887**	**8797042**	**A/G**	**0.379**	**0.116**	**99.5**
18	rs11706648	8796547	A/C	0.36	0.007	93.5
19	rs237885	8795543	T/G	0.426	<.05	50.8
20	rs2139184	8795494	C/A	0.008	1.0	100.0
**21**	**rs1042778**	**8794545**	**G/T**	**0.437**	**0.578**	**99.0**
22	rs6770632	8793724	C/A	0.394	<.05	68.8
23	rs9872310	8793381	A/G	0.138	0.035	100.0

Supplementary Table 2: Shows the haplotypes within the respective windows that differed significantly from the remaining ones. In the windows of sizes 5 to 2, with very few exceptions, haplotypes comprised of subsets of the SNPs of the 6-marker haplotypes drove the significant associations.

Window	rs237917	rs2268498	rs4564970	rs237897	rs2268495	rs53576	rs13316193	rs237889	rs2254298	rs2268494	rs9840864	rs237887	rs1042778
6	C	C	G	A	G	A							
	T	T	C	G	G	G							
5	C	C	G	A	G								
		C	G	A	G	A							
	T	T	C	G	G								
		T	C	G	G	G							
			C	G	G	G	T						
4		C	G	A	G								
			G	A	G	A							
		T	C	G	G								
			C	G	G	G							
			G	G	A	G							
3	C	C	G										
		C	G	A									
			G	A	G								
				A	G	A							
	T	T	C										
		T	C	G									
			C	G	G								
				G	A	G							
									A	T	C		
2	C	C											
		C	G										
			G	A									
				A	G								
	T	T											
		T	C										
			C	G									
				G	A								

Supplementary Table 3: Global p-value, permutation corrected global p-value (shown in brackets), and individual p-values for haplotypes of sizes 5 to 2. p-values for individual SNPs are shown at the bottom of the table.

	Haplotype	Frequency	Chi^2	p
	**rs237917 - rs2268498 - rs4564970 - rs237897 - rs2268495**			
	Likelihood ratio χ^2 = 14.74, df=5, global p-value = **0.012 (0.046)**			
	C-C-G-A-G	0.356	6.984	**0.008**
	C-T-G-G-G	0.214	0.043	0.835
	T-T-G-G-A	0.156	3.127	0.077
	T-T-C-G-G	0.076	6.519	**0.011**
	C-C-G-G-A	0.067	0.908	0.341
	T-T-G-A-G	0.054	0.845	0.358
	**rs2268498 - rs4564970 - rs237897 - rs2268495 – rs53576**			
	Likelihood ratio χ^2 = 15.29, df=6, global p-value = **0.018 (0.032)**			
	C-G-A-G-A	0.279	7.845	**0.005**
**Window 5**	T-G-G-G-G	0.213	0.072	0.788
	T-G-G-A-G	0.156	2.831	0.092
	T-C-G-G-G	0.089	7.270	**0.007**
	C-G-A-G-G	0.076	0.115	0.734
	C-G-G-A-G	0.067	0.954	0.329
	T-G-A-G-A	0.061	0.542	0.462
	**rs4564970 - rs237897 - rs2268495 – rs53576 – rs13316193**			
	Likelihood ratio χ^2 = 17.14, df=7, global p-value = **0.017 (0.043)**			
	G-A-G-A-T	0.290	5.565	**0.018**
	G-G-A-G-C	0.164	1.739	0.183
	G-G-G-G-T	0.155	0.047	0.829
	G-G-G-G-C	0.091	0.344	0.557
	C-G-G-G-T	0.070	7.432	**0.006**
	G-G-A-G-T	0.057	3.389	0.066
	G-A-G-G-C	0.056	1.003	0.317

Window	Haplotype	Frequency	Chi^2	p
Window 4	**rs2268498 - rs4564970 - rs237897 - rs2268495**			
	Likelihood ratio χ^2 = 16.13, df=5, global p-value = **0.006 (0.026)**			
	C-G-A-G	0.355	7.842	**0.005**
	T-G-G-G	0.218	0.001	0.973
	T-G-G-A	0.155	2.626	0.105
	T-C-G-G	0.091	7.551	**0.006**
	C-G-G-A	0.067	0.952	0.329
	T-G-A-G	0.066	1.467	0.226
	**rs4564970 - rs237897 - rs2268495 – rs53576**			
	Likelihood ratio χ^2 = 14.83, df=4, global p-value = **0.005 (0.031)**			
	G-A-G-A	0.347	7.630	**0.006**
	G-G-G-G	0.241	0.061	0.804
	G-G-A-G	0.217	4.136	**0.041**
	C-G-G-G	0.010	6.440	**0.011**
	G-A-G-G	0.082	0.488	0.485

Window	Haplotype	Frequency	Chi^2	p
Window 3	**rs237917 - rs2268498 - rs4564970**			
	Likelihood ratio χ^2 = 9.90, df=3, global p-value = **0.019 (0.021)**			
	C-C-G	0.458	4.888	**0.027**
	C-T-G	0.227	0.295	0.587
	T-T-G	0.210	3.178	0.075
	T-T-C	0.082	5.299	**0.021**
	**rs2268498 - rs4564970 – 237897**			
	Likelihood ratio χ^2 = 14.40, df=4, global p-value = **0.006 (0.030)**			
	T-G-G	0.374	1.665	0.197
	C-G-A	0.356	7.450	**0.006**
	C-G-G	0.10	0.270	0.603

	T-C-G	0.092	7.669	**0.006**
	T-G-A	0.066	1.368	0.242
	**rs4564970 – rs237897 – rs2268495**			
	Likelihood ratio χ^2 = 14.66, df=3, global p-value = **0.002 (0.005)**			
	G-A-G	0.421	9.805	**0.002**
	G-G-G	0.252	0.010	0.917
	G-G-A	0.223	3.867	**0.049**
	C-G-G	0.104	6.387	**0.012**
	**rs237897 – rs2268495 – rs53576**			
	Likelihood ratio χ^2 = 10.01 df=3, global p-value = **0.018 (0.023)**			
	G-G-G	0.344	1.866	0.170
	A-G-A	0.341	7.610	**0.006**
	G-A-G	0.223	4.104	**0.043**
	A-G-G	0.080	0.500	0.481
	**rs2254298 – rs2268494 – rs9840864**			
	Likelihood ratio χ^2 = 8.23 df=3, global p-value = **0.042 (0.046)**			
	G-T-G	0.771	0.336	0.562
	A-T-C	0.093	6.269	**0.012**
	G-T-C	0.070	2.386	0.122
	G-A-C	0.060	0.151	0.697
**Window 2**	**Haplotype**	**Frequency**	**Chi^2**	**p**
	**rs237917 - rs2268498**			
	Likelihood ratio χ^2 = 8.43, df=2, global p-value = **0.015 (0.012)**			
	C-C	0.469	5.493	**0.019**
	T-T	0.291	7.691	**0.006**
	C-T	0.241	0.039	0.844
	**rs2268498 - rs4564970**			
	Likelihood ratio χ^2 = 10.02, df=2, global p-value = **0.007 (0.021)**			

C-G	0.456	6.059	**0.014**
T-G	0.439	0.913	0.339
T-C	0.091	7.098	**0.008**

**rs4564970 - rs237897**			
Likelihood ratio χ^2 = 12.87, df=2, global p-value = **0.002 (0.007)**			
G-G	0.474	2.400	0.121
G-A	0.421	9.805	**0.002**
C-G	0.104	6.387	**0.012**

**rs237897 - rs2268495**			
Likelihood ratio χ^2 = 10.33, df=2, global p-value = **0.006 (0.003)**			
A-G	0.422	9.832	**0.002**
G-G	0.355	2.340	0.126
G-A	0.223	3.848	**0.050**

	rs number	MAF	Chi^2	p
**Single SNPs**	rs237917	0.296	7.026	**0.008**
	rs2268498	0.470	5.763	**0.017**
	rs4564970	0.106	9.832	**0.002**
	rs237897	0.432	3.863	**0.049**
	rs53576	0.216	5.980	**0.014**
	rs2254298	0.106	5.680	**0.017**
